# Impact of Nontreatment Duration and Keratopathy on Major Adverse Cardiovascular Events in Fabry Disease: A Nationwide Cohort Study

**DOI:** 10.3390/jcm13020479

**Published:** 2024-01-15

**Authors:** Aram Yang, Sinae Kim, Yong Jun Choi

**Affiliations:** 1 Department of Pediatrics, Kangbuk Samsung Hospital, Sungkyunkwan University School of Medicine, Seoul 03181, Republic of Korea; aram.yang@samsung.com; 2 Biostatistics Collaboration Team, Research Core Center, National Cancer Center, Goyang 10408, Republic of Korea; anacooll@naver.com; 3 Division of Pulmonary and Critical Care Medicine, Department of Internal Medicine, Gangnam Severance Hospital, Yonsei University College of Medicine, Seoul 04763, Republic of Korea

**Keywords:** cardiovascular diseases, comorbidity, Fabry disease, glycosphingolipids, risk factors

## Abstract

Fabry disease (FD) is a rare inherited X-linked lysosomal storage disorder that results in the progressive accumulation of glycosphingolipids in multiple organs. Early FD-specific treatments may improve clinical outcomes; however, clinical evidence about early FD treatment is limited. We aimed to determine the cardiovascular outcomes of patients with FD who received enzyme replacement therapy. This nationwide observational study was conducted using the National Health Claims database of the Korean population with FD. The primary outcome was major adverse cardiovascular events (MACEs). MACE risk factors in FD were evaluated using time-dependent Cox regression. Between January 2007 and April 2022, 188 patients with FD were analyzed. Among them, 22 (11.7%) experienced MACE (males: 14/95 [14.7%]; females: 8/93 [8.6%]). The mean age at MACE diagnosis was 53.5 ± 11.0 years in all patients with FD, which was lower in males compared with in females (49.7 ± 9.6 vs. 60.0 ± 10.7 years, *p* = 0.030). Multivariate analysis (HR, 95% CI) revealed that age (1.042; 1.004–1.082) and duration of FD nontreatment (1.040; 1.003–1.078) were significant MACE risk factors in all patients. In males, age (1.080; 1.032–1.131), FD nontreatment duration (1.099; 1.048–1.152), and keratopathy (18.920; 4.174–85.749) were significant MACE risk factors in multivariate analysis. In females, the only significant MACE risk factor was a high Charlson comorbidity index score (1.795; 1.229–2.622). In conclusion, duration of FD nontreatment and keratopathy are significant MACE risk factors in males with FD. These findings suggest the importance of early initiation of FD-specific treatment and careful evaluation of keratopathy in males with FD.

## 1. Introduction

Fabry disease (FD) is a lysosomal storage disorder associated with the accumulation of neutral glycosphingolipids and globotriaosylceramide (Gb3). It results from the decreased activity of alpha-galactosidase A (OMIM 301500) [1]. Gb3 accumulation results in multiorgan involvement, including the heart, brain, kidney, and eyes, leading to hypertrophy, fibrosis, and inflammation [2,3,4].

FD is an X-linked inherited disorder; males with FD are hemizygous and more severely affected as compared to females with FD. However, random or skewed (non-random) X-chromosome inactivation in females can lead to a more complicated clinical phenotype and prognosis. Therefore, symptom severity and organ involvement may vary depending on the organ or tissue in which the mutant gene is expressed in a significant proportion of cells [5,6].

Depending on manifestation type and onset, the phenotype of FD is classified into childhood and adolescence, early adulthood, and late adulthood [7] or classic and late-onset variants [8]. Although most patients remain asymptomatic during early life, “classic” FD leads to progressive multiorgan failure and premature death if left untreated.

Cardiovascular disease (CVD) is the primary cause of death in patients with FD and is associated with a significantly reduced life expectancy [9]. However, the risk factors and optimal timing for initiating enzyme replacement therapy (ERT) to prevent CVD in patients with FD remain unclear. According to Weidemann et al., initiating FD treatment before the development of myocardial fibrosis is recommended for achieving long-term improvements in myocardial morphology and function [10]. Similarly, Perera et al. suggested that early intervention with FD-specific therapies may improve clinical outcomes [11]. Nevertheless, larger and longer-term clinical studies are needed to provide further guidance on treatment. Moreover, previous studies enrolled and followed up patients with FD only after diagnosis, which limited the evaluation of pre-diagnosis comorbidities, progression of pre-treatment status, and changes in clinical manifestations.

Therefore, in this study, we aimed to evaluate the clinical manifestations and cardiovascular outcomes of FD using a large-scale nationwide population cohort over 15 years via time-dependent Cox regression.

## 2. Methods

### 2.1. Study Database

This study used the national health claims database established by the National Health Insurance Service (NHIS) of Korea. The NHIS is a mandatory health insurance system, covering approximately 97.1% (52 million) of the Korean population as National Health Insurance recipients. The NHIS database also includes information on medical aid recipients (the remaining 2.9% with low income), thus covering the entire Korean population. In addition, all patients with FD with relevant clinical symptoms, reduced alpha-galactosidase A activity on enzyme assay, and *GLA* gene mutation were registered in the rare intractable disease (RID) registry and assigned a code (V117 for FD).

The study was conducted according to the guidelines of the Declaration of Helsinki and approved by the Institutional Review Board of Kangbuk Samsung Hospital (IRB No. 2021-12-040). The requirement for informed consent was waived owing to the retrospective nature of the study using de-identified administrative data.

### 2.2. Study Participants

Between January 2007 and April 2022, patients with the International Classification of Diseases—Tenth Revision (ICD-10) diagnosis code E75.2 (other sphingolipidoses including E75.21, Fabry–Anderson disease; E75.22, Gaucher disease; E75.23, Krabbe disease; E75.24, Niemann–Pick disease) were identified (Supplementary Figure S1). To identify FD cases, patients who were prescribed specific FD drugs, including agalsidase beta (Fabrazyme, Fabagal), agalsidase alfa (Replagal), and migalastat (Galafold), were noted. Patients who had already received diagnostic or medication codes for FD before the study period and cases without the FD RID code (V117) were excluded.

### 2.3. Data Access and Collection

The NHIS databases are only available to researchers whose study protocols are approved by the official review committee.

Supplementary Table S1 describes the definitions of comorbidities and outcomes. Comorbidities were evaluated using the Charlson comorbidity index (CCI) and classified using Quan et al.’s [12] algorithm.

### 2.4. Outcomes

The primary study outcome was major adverse cardiovascular events (MACEs) that required hospitalization, defined as CVD (I00-I99)-related death, fatal or nonfatal myocardial infarction (MI) (I21-I23), and stroke (I60-I64) [13]. All-cause mortality was defined as in-hospital deaths and having no medical claim for over 1 year [14]. The number of outpatient department visits, number of hospital admissions, and total hospitalization time were also analyzed.

### 2.5. Statistical Analysis

Categorical variables are presented as frequencies (numbers) and percentages, and the chi-squared or Fisher’s exact test was used for comparison. Continuous variables are presented as mean ± standard deviation (SD) for normally distributed data or median (interquartile range) for non-normally distributed data and were compared using the Student’s *t*-test or Wilcoxon rank-sum test, respectively.

To estimate the risk factors for MACEs, time-dependent Cox regression models were used, which included time scales split into 2-week intervals between January 2007 and April 2022, resulting in a total of 66,963 timescales. All variables in the time-dependent analysis were evaluated at the beginning of each interval. Individuals were censored at the time of the MACE or study closure (April 2022). Univariable factors with *p* < 0.05 for MACE were included in the multivariate model. All analyses, including interaction tests, used a 95% significance level, and two-tailed *p* values < 0.05 were considered statistically significant.

All statistical analyses were performed using R (version 3.6.1, R Foundation, Vienna, Austria). Survival analyses and time-dependent Cox regression analysis were performed using the “survival” R package, and Kaplan–Meier curves were plotted using the “survminer” R package. The spline curves of the hazard ratio for MACE development were drawn using the “pspline” R package.

### 2.6. Data Availability

The data that support the findings of this study are available from the National Health Insurance Sharing Service (NHISS, https://nhiss.nhiss.or.kr/, accessed on 2 May 2022). However, restrictions apply regarding the availability of the data, which were used with permission for the present study and, therefore, are not publicly available. However, they may be made available through the corresponding author upon reasonable request and permission from the NHISS.

## 3. Results

### 3.1. Characteristics of Patients with Fabry Disease

Between January 2007 and April 2022, 209 individuals in the Korean population were diagnosed with the E75.2 ICD 10 code and received FD-specific treatments (Supplementary Figure S1). After excluding individuals diagnosed and treated for FD before 2007, 188 (95 males and 93 females) patients with FD were included in the final analysis without follow-up loss.

**Figure 1:**
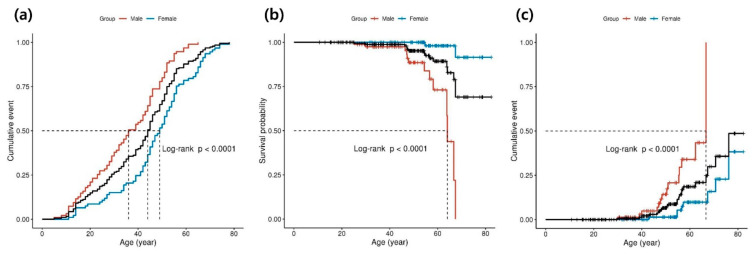
Kaplan–Meier curves of diagnosis, all-cause mortality, and major adverse cardiovascular events in Fabry disease. The *p*-value for comparing the two groups was calculated using the log-rank test. (**a**) Diagnostic probability in patients with Fabry disease. The *x*-axis represents the time since birth (years), and the *y*-axis represents the cumulative Fabry disease incidence in the total cohort (black) and subcohorts of males (red) and females (blue). (**b**) Cumulative all-cause mortality in patients with Fabry disease. The *x*-axis represents the time since birth (years), and the *y*-axis represents the survival probability mortality in the total cohort (black) and subcohorts of males (red) and females (blue). (**c**) Cumulative incidence of MACEs in patients with Fabry disease. The *x*-axis represents the time since birth (years), and the *y*-axis represents the cumulative MACEs in the total cohort (black) and subcohorts of males (red) and females (blue). MACEs; major adverse cardiac events.

Compared with female patients, a significantly higher proportion of male patients were diagnosed with FD before the age of 30 years. The mean age at diagnosis for all patients was significantly higher in females (47.6 ± 15.7 vs. 35.8 ± 15.1, *p* < 0.001; Table 1). The difference in age at diagnosis and treatment initiation was significantly greater in females. Kaplan–Meier analysis showed that FD diagnosis was made significantly earlier in males than in females (median diagnostic probability: 36.0 vs. 49.0 years, *p* < 0.001 by log-rank test; Figure 1a).

When comparing comorbidities at the time of FD diagnosis, there was no significant difference in CCI scores between males and females; however, renal disease was more prevalent in males in the CCI subcategories, with end-stage renal disease (ESRD) being significantly higher in males. Table 1 presents other diagnostic characteristics and comorbidities of patients with FD.

### 3.2. Clinical Outcomes and MACEs in Fabry Disease

FD-specific treatment with agalsidase alfa, agalsidase beta, and migalastat was administered to 188 patients. No significant differences were observed in therapeutic characteristics between males and females. Regarding clinical outcomes, no significant differences were observed in the number of outpatient clinic visits, admissions, or total length of hospitalization between males and females. However, all-cause mortality was higher in males (Table 2). Kaplan–Meier analysis showed that male patients with FD had higher all-cause mortality than female patients with FD (median survival, 64.1 years vs. not reached; *p* < 0.001, log-rank test; Figure 1b).

Over 15 years, MACEs occurred in 22/188 (11.7%) patients, of which 14/95 were males (14.7%), whereas 8/93 were females (8.6%). The mean age at MACE diagnosis was 53.5 ± 11.0 years in all patients with FD and was lower in males (49.7 ± 9.6 vs. 60.0 ± 10.7 years, *p* = 0.030). Specifically, the age at MI diagnosis was significantly higher in males (46.3 ± 8.1 years) than in females (73.5 ± 3.9 years) (*p* = 0.012). In the Kaplan–Meier analysis, male patients with FD demonstrated a higher cumulative MACE (median, 66.9 years vs. not reached, *p* < 0.001 by log-rank test, Figure 1c).

Concerning comorbidities, ESRD was significantly more prevalent in males than in females (26.3% vs. 2.2%, *p* < 0.001). Although the CCI score and prevalence of hypertension, heart failure, chronic kidney disease (CKD), DM, dyslipidemia, and keratopathy were not significantly different between males and females, these comorbidities developed earlier in males than in females (Table 2 and Table S2).

There was no sex difference between the MACE and non-MACE groups. However, the non-MACE group showed the use of agalsidase beta more frequently than the MACE group (94.0% vs. 68.2%, respectively, *p* < 0.001). There were significant differences in clinical outcomes between the two groups. The MACE group showed a significantly higher total number of outpatient clinic visits and admissions, total hospitalization duration, and all-cause mortality compared with the non-MACE group (median [1st quartile–3rd quartile]; 225.0 [136.0–375.0] vs. 176.5 [96.0–285.0] times, *p* = 0.035; 20.5 [6.0–40.0] vs. 7.0 [3.0–18.0] times, *p* = 0.005; 299.5 [118.0–529.0] vs. 68.0 [28.0–168.0] days, *p* < 0.001; and 40.9% vs. 3.6%, *p* < 0.001, respectively). In terms of comorbidities, the MACE group had significantly higher CCI scores and prevalence of CKD, ESRD, and DM compared with those in the non-MACE group.

### 3.3. Risk Factors for MACEs in Fabry Disease

In univariate analyses, the risk factors for MACEs were age, duration of FD nontreatment, CCI score, peripheral vascular disease, mild liver disease, malignancy, DM, CKD, and ESRD. In multivariate analyses, age and duration of FD nontreatment were MACE risk factors (Table 3).

A subgroup analysis according to sex was performed to account for the differences in disease progression between males and females.

In univariate analyses, the risk factors for MACEs in male patients were age, nontreatment duration, CCI, peripheral vascular disease, malignancy, keratopathy, DM, CKD, and ESRD. In the multivariate analysis, age, nontreatment duration, and keratopathy were significant MACE risk factors in male patients. Notably, keratopathy was the most significant factor for MACEs in males.

**Figure 2:**
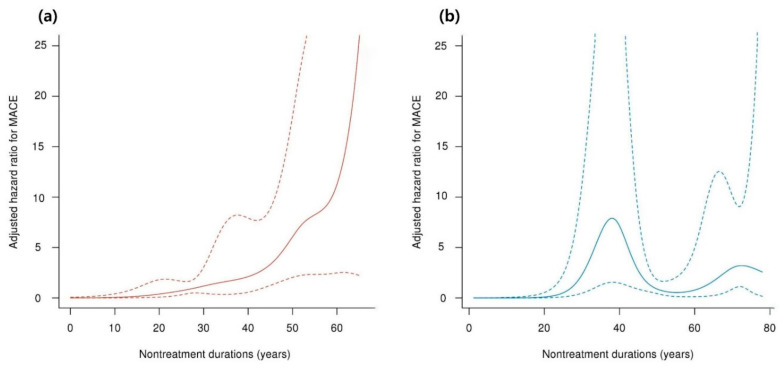
Correlation of nontreatment duration and adjusted hazard ratio for MACEs. (**a**) Male and (**b**) female patients with Fabry disease. The *x*-axis represents the nontreatment duration (in years), and the *y*-axis represents the adjusted HR for MACEs in males (**a**) and females (**b**). The solid line represents the HR, whereas the dotted line represents the 95% confidence interval. Abbreviations: HR, hazard ratio; MACE, major adverse cardiovascular event.

In females, the following factors were identified as MACE risk factors in univariate analysis: age, nontreatment duration, CCI score, peptic ulcer disease, mild liver disease, DM, CKD, and ESRD. However, in multivariate analysis, only a high CCI score was a significant MACE risk factor in females. Additionally, when considering nontreatment duration, the HR curve demonstrated divergent patterns between males and females. In males, the adjusted HR increased with longer nontreatment duration, whereas in females, no consistent correlation was observed between adjusted HR and nontreatment duration (as illustrated in Figure 2a,b, respectively).

### 3.4. Clinical Impact of Keratopathy in Fabry Disease

Male and female patients showed no significant difference in the development of keratopathy. Additionally, amiodarone use did not differ between the non-keratopathy and keratopathy groups (7/144 [4.8%] vs. 4/44 [9.0%], *p* = 0.295). Supplementary Table S3 compares patient characteristics according to the presence of keratopathy, stratified by sex. Male patients with keratopathy experienced congestive heart failure significantly earlier than those without (35.3 ± 7.8 vs. 45.1 ± 13.1 years, *p* = 0.050). However, no significant difference was observed among female patients (50.5 ± 9.3 vs. 50.0 ± 14.0 years, *p* = 0.908, respectively).

**Figure 3:**
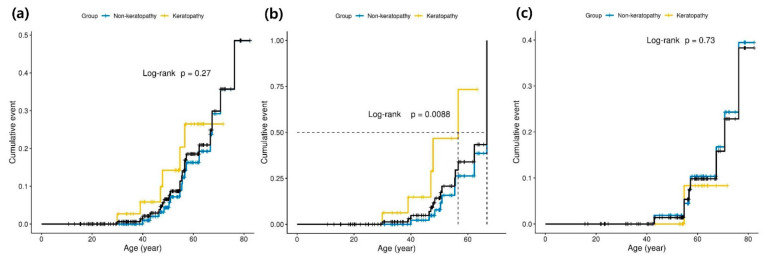
Cumulative incidence of MACEs in Fabry disease according to keratopathy stratified by sex. (**a**) Comparison within the total cohort (black line). (**b**) Comparison within a group of male patients (black line). (**c**) Comparison within a group of female patients (black line). Within each cohort, the group with keratopathy is marked with a yellow line, and the group without keratopathy is marked with a blue line. Event rates were estimated using the Kaplan–Meier method, and comparisons were made using the log-rank test. Abbreviations: MACE, major adverse cardiovascular event.

Kaplan–Meier analysis showed no significant difference in MACE incidence between patients with and without keratopathy in the overall population (median cumulative MACE; not reached vs. not reached, *p* = 0.270 by log-rank test, Figure 3a). However, in the subgroup analysis based on sex, males with keratopathy had a significantly higher MACE incidence than those without (median cumulative MACE; 56.5 vs. 66.5 years, *p* = 0.009 by log-rank test, Figure 3b). Females with and without keratopathy showed no significant difference in MACE incidence (median cumulative MACE; not reached vs. not reached, *p* = 0.730 by log-rank test, Figure 3c).

## 4. Discussion

This retrospective, nationwide, population-based cohort study investigated the relationship between Fabry disease (FD) nontreatment duration, age, and the risk of major adverse cardiovascular events (MACEs) in a cohort of 188 patients with FD over a 15-year period. The results revealed sex-specific differences in MACE risk factors, which may be related to the X-linked genetic nature of the disease. For male patients, FD nontreatment duration and keratopathy were identified as key MACE risk factors. However, for female patients, the presence of other comorbidities was a significant MACE risk factor, likely owing to the slower progression of FD. These findings highlight the importance of sex-specific risk factors in managing and treating FD.

MACE exhibits variations in its definition and components across studies, reflecting diverse outcomes based on race and country. Kim et al. reported that the average onset age of MI in Koreans is 65.9 ± 13.0 years, with males at 63.0 ± 12.4 years and females at 74.0 ± 11.0 years [15]. Another study by Kim et al. reported similar average onset ages of MI [16]. In this study, the average onset ages of MI in total, male, and female FD patients were 55.4 ± 15.5, 46.3 ± 8.1, and 73.5 ± 3.9, respectively. In females, there was no difference between the general population and FD patients, whereas males with FD showed an earlier onset age of MI compared with the general population. In both the general population and FD patients, MI showed a higher prevalence among males [15,16,17]. Regarding stroke, Ryu et al. reported the average onset age of stroke at 67.5 ± 12.6 years, with males at 65.1 years and females at 71.1 years [18]. Kim et al. also reported the mean age as 64.8 years in males and 70.1 years in females [19]. In our study of FD patients, the onset age of stroke was 52.8 ± 6.0 years in overall FD patients, 52.7 years in male FD patients, and 52.8 years in female FD patients. This indicates that both male and female FD patients experienced stroke earlier than the general population. However, unlike the general population, no sex difference in the occurrence of stroke was observed among FD patients. Similarly, the Fabry Registry reported a distinct pattern of stroke occurrence compared with the general population [20]. While challenging to explain, Kolodny et al. suggested that this could be attributed to differences in compensating for the physiological effects of GL-3 accumulation [21]. Considering the variations in study designs, comparative research between the general population and Fabry patients is necessary to account for these differences in results.

In patients with FD, MACE is a critical prognostic factor. However, few studies have identified MACE risk factors or predictors and the optimal timing to initiate FD-specific treatment to prevent MACE. Although research on the efficacy of ERT in preventing MACEs is inadequate, several studies have explored FD cardiomyopathy. Weidemann et al. showed that agalsidase alfa enhances myocardial morphology and functions in FD cardiomyopathy and is a recommended early treatment before myocardial fibrosis development [10]. Hughes et al. also reported that ERT regresses hypertrophic cardiomyopathy in FD [22]. Kampmann et al. presented the effect of agalsidase alfa treatment over 10 years in controlling progression and improving symptoms of FD cardiomyopathy [23]. The findings of the current study are consistent with those of previous research, highlighting the significance of early treatment to prevent MACEs in males. However, in female patients with FD, the adjusted analysis showed no significant association between FD nontreatment duration and MACEs, indicating the importance of controlling comorbidities associated with high CCI scores. Similarly, a recent study reported differences in MACE according to sex in FD, emphasizing the importance of individualized sex-specific follow-up and treatment [24]. Further research is necessary to address this issue.

Fabry keratopathy, also known as cornea verticillata, is characterized by the accumulation of lipid inclusions of unmetabolized Gb3 within the cornea and is more common in males and females with classic FD [25,26,27]. The prevalence of Fabry keratopathy is 50–94% in adult patients with classic FD. In a systematic review and meta-analysis of 460 records, Van Der Tol et al. reported a 69% pooled prevalence of Fabry keratopathy (74% in males and 66% in females) in adult patients with classic FD. Although a study suggested that long-term ERT improves corneal changes [26], the effect of ERT on Fabry keratopathy remains unestablished.

Several studies suggest that Fabry keratopathy indicates severe disease with a high Fabry Outcome Survey–Mainz Severity Score Index (FOS-MSSI) [28,29]. The total FOS-MSSI score is an index of FD severity consisting of four domains: general, neurologic, cardiovascular, and renal. Each domain is assigned a weight based on its impact on disease morbidity [30]. An ocular expression analysis of FD in 173 adult patients included in the FOS revealed a correlation between tortuous ocular vessels, high FOS-MSSI values, and renal and cardiac dysfunction, indicating that ocular manifestations are an early marker of systemic disease in the FD [26]. The current study concurs with previous research by adjusting time-weighted variables at each time point and analyzing MACE risk factors in each patient over time.

Previous studies had limitations, such as a small sample size, a brief follow-up period, and limited data prior to FD diagnosis. Our study complements previous results and emphasizes the importance of evaluating keratopathy. While keratopathy does not directly cause MACEs, it can serve as a MACE predictor and an indicator for initiating treatment in FD. However, globally, ocular signs, including Fabry keratopathy, are not considered indicators for FD-specific ERT. Therefore, they are not recommended as initial assessment items for FD [31,32,33,34,35,36]. Additionally, in the latest Health Services Executive guideline for FD in 2022, ocular symptoms are overlooked, and treatment initiation is only considered in later-onset patients with FD with renal, cardiac, gastrointestinal, or general symptoms (e.g., significant pain) [37,38]. Thus, further research and consensus are necessary to address these issues.

Amiodarone has been associated with keratopathy [39,40]; therefore, caution is advised when considering the use of amiodarone in patients with FD, although there is insufficient research and evidence to reach a consensus [1,41]. In this study, patients with FD with or without keratopathy exhibited no significant difference in amiodarone use. Therefore, keratopathy should be evaluated differently in patients with FD compared to in general patients.

This study had some limitations. First, confounding factors that were not included in the study may have affected the results. Specifically, lifestyle factors known to influence MACE occurrence, such as habits related to smoking, diet, and exercise, were not included as variables in this study. The sexual differences in these variables may have impacted the results of this study. In addition, multiple MACEs were not considered in this study, which may also have acted as a confounding factor. Second, keratopathy was not directly evaluated through a slit lamp but through ophthalmologic diagnostic codes, possibly affecting the results. Third, with an observation period exceeding 15 years, variations in individual patient observation periods could influence the results. We utilized time-dependent Cox regression to minimize this limitation and account for variables changing over an extended duration. Finally, as FD is rare, a limited number of patients were included in the study, which may have affected the significance of some results.

The study has several strengths that enhance the significance of the results. To the best of our knowledge, this is the first study to identify MACE risk factors in patients with FD and directly demonstrate the relationship between MACE, untreated patients, and keratopathy. Second, the study included a relatively large sample size of 188 patients over an extended observation period of 15 years, which is a strength compared to previous FD-related studies. Third, as the study only included patients who received FD-specific treatment, it allowed for a more direct comparison of treatment effects in individual patients. Additionally, the study was a national cohort research of patients with a rare disease using big data and followed patients from pre-diagnosis to the end of the observation period, minimizing recall bias based on objective claims data. Finally, the study analyzed the time-dependent course of the disease using time-dependent Cox regression, providing more accurate results in evaluating the effects of each variable over time.

## 5. Conclusions

In conclusion, the duration of FD nontreatment and keratopathy are significant risk factors for MACE, particularly in male patients with FD. Thus, early initiation of FD-specific treatment and careful evaluation of keratopathy are crucial for male patients with FD. Contrastingly, these factors were not significant contributors to MACE in female patients. Instead, the presence of other comorbidities emerged as a critical risk factor. This difference may be attributed to the X-linked inheritance of Fabry disease; however, additional large-scale studies are required for confirmation.

## Figures and Tables

**Table 1:** Baseline characteristics of patients at the time of diagnosis for Fabry disease.

	Total (n = 188)	Male (n = 95)	Female (n = 93)	*p*
Type				0.002 *
Classic (age at diagnosis < 30 years)	48 (25.5%)	34 (35.8%)	14 (15.1%)	
Atypical (age at diagnosis ≥ 30 years)	140 (74.5%)	61 (64.2%)	79 (84.9%)	
Age at diagnosis, years	41.6 ± 16.4	35.8 ± 15.1	47.6 ± 15.7	<0.001 *
Age at start of treatment, years	42.2 ± 16.4	36.2 ± 14.9	48.4 ± 15.5	<0.001 *
Difference in age between diagnosis and treatment	0.6 ± 1.3	0.4 ± 1.0	0.8 ± 1.5	0.025 *
Comorbidities at diagnosis				
CCI (score)	2.0 ± 2.5	2.4 ± 2.9	2.1 ± 2.1	0.360
Myocardial infarction	17 (9.0%)	9 (9.5%)	8 (8.6%)	1.000 ^†^
Congestive heart failure	45 (23.9%)	18 (18.9%)	27 (29.0%)	0.147
Peripheral vascular disease	20 (10.6%)	11 (11.6%)	9 (9.7%)	0.852
Cerebrovascular disease	26 (13.8%)	14 (14.7%)	12 (12.9%)	0.879
Dementia	2 (1.1%)	2 (2.1%)	0 (0.0%)	0.497 ^†^
Chronic pulmonary disease	69 (36.7%)	30 (31.6%)	39 (41.9%)	0.186
Rheumatic disease	12 (6.4%)	6 (6.3%)	6 (6.5%)	1.000 ^†^
Peptic ulcer disease	56 (29.8%)	22 (23.2%)	34 (36.6%)	0.064
Mild liver disease	64 (34.0%)	31 (32.6%)	33 (35.5%)	0.796
Diabetes without chronic complication	46 (24.5%)	21 (22.1%)	25 (26.9%)	0.554
Diabetes with chronic complication	3 (1.6%)	2 (2.1%)	1 (1.1%)	1.000 ^†^
Hemiplegia or paraplegia	4 (2.1%)	2 (2.1%)	2 (2.2%)	1.000 ^†^
Renal disease	36 (19.1%)	28 (29.5%)	8 (8.6%)	0.001 *^†^
Any malignancy ^‡^	8 (4.3%)	3 (3.2%)	5 (5.4%)	0.695
Moderate or severe liver disease	1 (0.5%)	1 (1.1%)	0 (0.0%)	1.000 ^†^
Metastatic solid tumor	2 (1.1%)	2 (2.1%)	0 (0.0%)	0.497 ^†^
HIV/AIDS	0 (0.0%)	0 (0.0%)	0 (0.0%)	1.000 ^†^
Hypertension	88 (46.8%)	45 (47.4%)	43 (46.2%)	0.993
Heart failure	90 (47.9%)	45 (47.4%)	45 (48.4%)	1.000
Chronic kidney disease	49 (26.1%)	30 (31.6%)	19 (20.4%)	0.115
End-stage renal disease	18 (9.6%)	17 (17.9%)	1 (1.1%)	<0.001 *^†^
Diabetes mellitus	51 (27.1%)	24 (25.3%)	27 (29.0%)	0.677
Dyslipidemia	87 (46.3%)	43 (45.3%)	44 (47.3%)	0.892
MACEs	6 (3.2%)	5 (5.3%)	1 (1.1%)	0.223
Cardiovascular disease-related death	0 (0.0%)	0 (0.0%)	0 (0.0%)	1.000 ^†^
Myocardial infarction	3 (1.6%)	3 (3.2%)	0 (0.0%)	0.246 ^†^
Stroke	3 (1.6%)	2 (2.1%)	1 (1.1%)	1.000 ^†^
Keratopathy	4 (2.1%)	3 (3.2%)	1 (1.1%)	0.628

Data are presented as n (%) or mean ± standard deviation. All continuous values were assessed for *p*-values using the *t*-test. *p*-values marked with ^†^ were obtained using Fisher’s exact test, whereas the others were determined using the chi-squared test. ^‡^ Including lymphoma and leukemia, except malignant neoplasm of the skin. CCI, Charlson comorbidity index; HIV/AIDS, human immunodeficiency virus/acquired immunodeficiency syndrome; MACEs, major adverse cardiovascular events. * *p* < 0.05.

**Table 2:** Clinical outcomes and comorbidities in patients with Fabry disease.

	Total (n = 188)	Male (n = 95)	Female (n = 93)	*p*	Non-MACE (n = 166)	MACE (n = 22)	*p*
Sex (male)					81 (48.8%)	14 (63.6%)	0.280
Type of treatment							
Agalsidase alfa	29 (15.4%)	13 (13.7%)	16 (17.2%)	0.641	29 (17.5%)	0 (0.0%)	0.069 ^†^
Agalsidase beta	171 (91.0%)	86 (90.5%)	85 (91.4%)	1.000 ^†^	156 (94.0%)	15 (68.2%)	<0.001 *
Migalastat	11 (5.9%)	7 (7.4%)	4 (4.3%)	0.558	11 (6.6%)	0 (0.0%)	0.447 ^†^
Clinical outcomes							
Outpatient visits, n	178.0 [101.0–294.5]	180.0 [113.5–312.0]	178.0 [94.0–257.0]	0.154	176.5 [96.0–285.0]	225.0 [136.0–375.0]	0.035 *
Admissions, n	8.0 [3.0–19.0]	10.0 [4.0–23.0]	6.0 [3.0–16.0]	0.168	7.0 [3.0–18.0]	20.5 [6.0–40.0]	0.005 *
Total admissions, days	80.0 [32.0–205.0]	94.0 [33.5–246.0]	75.5 [32.0–152.0]	0.202	68.0 [28.0–168.0]	299.5 [118.0–529.0]	<0.001 *
All-cause mortality, n	15 (8.0%)	13 (13.7%)	2 (2.2%)	0.008 *	6 (3.6%)	9 (40.9%)	<0.001 *
Age at death, years	53.1 ± 12.9	51.9 ± 13.3	61.1 ± 9.0	0.370	50.8 [47.1–63.8]	56.6 [47.8–66.7]	0.388
Total MACEs	22 (11.7%)	14 (14.7%)	8 (8.6%)	0.280			
Onset age, years	53.5 ± 11.0	49.7 ± 9.6	60.0 ± 10.7	0.030 *			
CV-related death	7 (3.7%)	5 (5.3%)	2 (2.2%)	0.458			
Onset age, years	52.8 ± 13.0	49.5 ± 13.7	61.1 ± 9.0	0.331			
Myocardial infarction	6 (3.2%)	4 (4.2%)	2 (2.2%)	0.698			
Onset age, years	55.4 ± 15.5	46.3 ± 8.1	73.5 ± 3.9	0.012 *			
Stroke	9 (4.8%)	5 (5.3%)	4 (4.3%)	1.000 ^†^			
Onset age, years	52.8 ± 6.0	52.7 ± 6.3	52.8 ± 6.7	0.985			
Comorbidities at the end of the observation							
CCI, score	3.0 [2.0–6.0]	3.0 [2.0–6.0]	3.0 [2.0–5.0]	0.801	3.0 [2.0–5.0]	7.0 [6.0–8.0]	<0.001 *
Hypertension	144 (76.6%)	71 (74.7%)	73 (78.5%)	0.663	124 (74.7%)	20 (90.9%)	0.156
Onset age, years	43.6 ± 12.7	38.1 ± 10.9	48.9 ± 12.0	<0.001 *	43.0 ± 12.9	47.1 ± 10.8	0.176
Heart failure	153 (81.4%)	74 (77.9%)	79 (84.9%)	0.292	132 (79.5%)	21 (95.5%)	0.130
Onset age, years	43.4 ± 12.8	38.1 ± 11.3	48.3 ± 12.1	<0.001 *	42.6 ± 12.9	48.1 ± 11.3	0.068
Chronic kidney disease	77 (41.0%)	42 (44.2%)	35 (37.6%)	0.442	60 (36.1%)	17 (77.3%)	0.001 *
Onset age, years	45.4 ± 11.2	41.8 ± 9.7	49.8 ± 11.4	0.001 *	44.4 ± 10.6	49.0 ± 12.9	0.140
End-stage renal disease	27 (14.4%)	25 (26.3%)	2 (2.2%)	<0.001 *^†^	17 (10.2%)	10 (45.5%)	<0.001 *
Onset age, years	44.4 ± 10.2	43.8 ± 9.3	51.0 ± 23.0	0.735	41.8 ± 9.2	48.8 ± 10.8	0.087
Diabetes mellitus	95 (50.5%)	53 (55.8%)	42 (45.2%)	0.190	78 (47.0%)	17 (77.3%)	0.015 *
Onset age, years	44.5 ± 12.8	40.6 ± 11.9	49.3 ± 12.3	0.001 *	42.9 ± 12.9	51.6 ± 9.4	0.011 *
Dyslipidemia	154 (81.9%)	75 (78.9%)	79 (84.9%)	0.379 ^†^	133 (80.1%)	21 (95.5%)	0.144
Onset age, years	43.4 ± 14.4	38.0 ± 12.6	48.6 ± 14.3	<0.001 *	42.5 ± 14.7	49.6 ± 10.9	0.035 *
Keratopathy	44 (23.4%)	21 (22.1%)	23 (24.7%)	0.800	38 (22.9%)	6 (27.3%)	0.851
Onset age, years	40.7 ± 16.1	31.8 ± 13.9	48.8 ± 13.8	<0.001 *	40.6 ± 16.9	40.9 ± 11.2	0.971

Data are presented as n (categorical value, %), mean ± standard deviation (parametric continuous value), or median [1st quartile–3rd quartile (non-parametric continuous value)]. *p*-values marked with ^†^ were obtained using Fisher’s exact test, whereas the others were determined using the chi-squared test. *p*-values for parametric continuous values were assessed using the *t*-test, whereas non-parametric values were evaluated using the Wilcoxon rank-sum test. CV, cardiovascular; CCI, Charlson comorbidity index; MACEs, major adverse cardiovascular events. * *p* < 0.05.

**Table 3:** Time-dependent Cox regression analysis of risk factors for MACEs.

	Univariate Analysis		Multivariate Analysis		
	HR (95% CI)	*p*	HR (95% CI)	*p*	VIF
**Total patients**					
Age (years)	1.047 (1.024–1.071)	<0.001 *	1.042 (1.004–1.082) ^†^	0.028 *	
Nontreatment duration (years)	1.045 (1.022–1.068)	<0.001 *	1.040 (1.003–1.078)	0.035 *	1.520
Treatment duration (years)	0.999 (0.859–1.162)	0.990			
Difference in diagnosis and treatment (years)	0.839 (0.478–1.473)	0.540			
Female (vs. male)	0.603 (0.248–1.469)	0.266			
Comorbidities (CCI score)	1.344 (1.202–1.502)	<0.001 *	1.222 (0.937–1.594)	0.139	6.490
Peripheral vascular disease	3.217 (1.216–8.508)	0.019 *	0.957 (0.256–3.583)	0.948	2.229
Peptic ulcer disease	1.829 (0.794–4.209)	0.156			
Mild liver disease	7.226 (1.914–27.275)	0.004 *	2.063 (0.384–11.076)	0.398	2.340
Any malignancy ^†^	4.593 (1.502–14.047)	0.008 *	1.079 (0.124–9.355)	0.945	3.037
Keratopathy	2.555 (0.949–6.884)	0.063	5.845 (2.059–16.596) ^‡^	<0.001 *	
Diabetes mellitus	4.977 (1.517–16.333)	0.008 *	1.160 (0.262–5.130)	0.845	2.344
Chronic kidney disease	6.388 (2.566–15.907)	<0.001 *	2.550 (0.749–8.688)	0.134	1.805
End-stage renal disease	7.300 (3.087–17.265)	<0.001 *	1.614 (0.355–7.332)	0.535	2.194
**Male patients**					
Age (years)	1.088 (1.043–1.136)	<0.001 *	1.080 (1.032–1.131) ^†^	0.001 *	
Nontreatment duration (years)	1.086 (1.036–1.137)	0.001 *	1.099 (1.048–1.152)	<0.001 *	1.853
Treatment duration (years)	0.956 (0.803–1.137)	0.611			
Difference in diagnosis and treatment	0.827 (0.480–1.427)	0.496			
Comorbidities (CCI score)	1.253 (1.119–1.403)	<0.001 *	1.145 (0.805–1.629)	0.451	8.952
Peripheral vascular disease	4.161 (1.186–14.600)	0.026 *	2.687 (0.518–13.932)	0.239	2.092
Peptic ulcer disease	1.316 (0.398–4.352)	0.653			
Mild liver disease	3.802 (0.950–15.206)	0.059			
Any malignancy ^†^	6.353 (1.751–23.057)	0.005 *	0.786 (0.094–6.594)	0.824	3.322
Keratopathy	3.385 (1.042–10.999)	0.043 *	18.920 (4.174–85.749)	<0.001 *	2.316
Diabetes mellitus	3.597 (1.108–11.677)	0.033 *	0.866 (0.145–5.175)	0.875	3.804
Chronic kidney disease	7.755 (2.465–24.402)	<0.001 *	2.610 (0.465–14.640)	0.276	2.524
End-stage renal disease	6.684 (2.463–18.136)	<0.001 *	2.092 (0.639–6.848)	0.223	1.704
**Female patients**					
Age (years)	1.053 (1.012–1.097)	0.012	1.029 (0.971–1.090) ^†^	0.330	
Nontreatment duration (years)	1.048 (1.010–1.088)	0.013	1.014 (0.958–1.074)	0.630	2.222
Treatment duration (years)	1.065 (0.792–1.432)	0.676			
Difference in diagnosis and treatment (years)	0.906 (0.437–1.879)	0.791			
Comorbidities (CCI score)	2.073 (1.700–2.527)	<0.001 *	1.795 (1.229–2.622)	0.002 *	3.942
Peripheral vascular disease	2.285 (0.489–10.682)	0.294			
Peptic ulcer disease	4.247 (1.192–15.139)	0.026 *	3.156 (0.533–18.693)	0.205	1.275
Mild liver disease	23.558 (1.877–295.66)	0.014 *	2.427 (0.093–63.599)	0.595	2.710
Any malignancy ^†^	2.679 (0.291–24.670)	0.384			
Keratopathy	0.942 (0.100–8.910)	0.958	0.523 (0.104–2.629) ^‡^	0.431	
Diabetes mellitus	10.006 (1.028–97.428)	0.047 *	2.604 (0.08–84.694)	0.590	1.688
Chronic kidney disease	4.697 (1.138–19.381)	0.032 *	1.592 (0.242–10.45)	0.628	1.930
End-stage renal disease	15.130 (1.301–175.911)	0.030 *	2.655 (0.466–15.138)	0.271	1.537

Data are presented as n (%) or mean ± standard deviation. HR, hazard ratio; CI, confidence interval; VIF, variance inflation factors; CCI, Charlson comorbidity index. * *p* < 0.05 ^†^ Including lymphoma and leukemia, except malignant neoplasm of skin ^†^ Owing to multicollinearity with nontreatment duration, we analyzed the data using a multivariate model that omitted nontreatment duration. ^‡^ Although keratopathy did not show significance in univariate analysis of overall and female patients, we conducted a separate analysis by adding keratopathy to the existing multivariate model to investigate its significance in the overall patient as well as in male and female subgroups.

## Data Availability

The data that support the findings of this study are available from the National Health Insurance Sharing Service (NHISS, https://nhiss.nhis.or.kr/, accessed on 2 May 2022). However, restrictions apply regarding the availability of the data, which were used with permission for the present study and, therefore, are not publicly available. However, they may be made available through the corresponding author upon reasonable request and permission from the NHISS.

## Supplementary Materials

The following supporting information can be downloaded at: https://www.mdpi.com/article/10.3390/jcm13020479/s1, Figure S1: Study flowchart; Table S1: Definitions of comorbidities and outcomes; Table S2: Comparison of Charlson Comorbidity Index between males and females; Table S3: Comparison between patients with Fabry disease with and without keratopathy.
