# Supplementary material for: Impact of Nontreatment Duration and Keratopathy on Major Adverse Cardiovascular Events in Fabry Disease: A Nationwide Cohort Study
Source: J Clin Med. 2024 Jan 15;13(2):479. doi: 10.3390/jcm13020479 (PMC10817061; doi:10.3390/jcm13020479)
Supplement: Supplementary file 1 [file jcm-13-00479-s001.zip › jcm-2803641-supplementary.pdf]

Impact of Non-treatment Duration and Keratopathy on Major Adverse Cardiovascular Events in Fabry Disease

Aram Yang, Sinae Kim, Yong Jun Choi

Supplementary Table S1. Definitions of comorbidities and outcomes

Variables	Category	Code	Drug name (Main Ingredient Codes)	Elaboration	References
**MACE**					
Cardiovascular disease related death	ICD-10	I00-I99		Main or sub-diagnosis in-hospitalization	PMID 29118034, 26365022, 30882239, 34742250, 33739126, 31937196
Myocardial infarction	ICD-10	I21-23		Main or sub-diagnosis in-hospitalization	
Stroke (Haemorrhagic or ischemic)	ICD-10	I60-64		Main or sub-diagnosis in-hospitalization	
**Cardiac comorbidity**					
Hypertension	ATC	C02	clonidine (136505ATR), doxazosin (149102ATB,149104ATR), hydralazine (170701ATB), minoxidil (196102ATB), bosentan (485201ATB, 485202ATB), ambrisentan (564701ATB, 564702ATB), macitentan (632201ATB), hydralazine (170730BIJ), nitroprusside (229330BIJ)	2 or more categories of ATC 2/3/7/8/9/10 or ICD 10 code	https://www.whocc.no./ (Guidelines for ATC classification and DDD assignment 2022)
		C03	amiloride (106901ATB), sulfonamides (112901ATB), furosemide (163801ATB, 163830BIJ), hydrochlorothiazide (170801ATB), indapamide (174403ATB), spironolactone (231101ATB, 231102ATB), torasemide (242001ATB, 242002ATB, 242003ATB), xipamide (249401ATB), chlortalidone (451302ATB), indapamide (174401ATR), hydrochlorothiazide and potassium-sparing agents (262700ATB), tolvaptan (616501ATB,		PMID 32416785, 30742667

	616502ATB, 616503ATB)
C04	naftidrofuryl (198601ACH, 198601ATB), other peripheral vasodilators (172701ACR), ifenprodil (173201ATB), kallidinogenase (179303ATE), other peripheral vasodilators (184101ATB), nicergoline (201201ATB, 201203ATB, 201204ATB), pentoxifylline (210802ATR), phentolamine (441430BIJ)
C07	bisoprolol (117903ATB, 117904ATB), Alpha- and beta-blocking agents (110201ATB, 110202ATB), carteolol (124801ATB), propranolol (219901ATB, 219904ATB), sotalol (230402ATB), atenolol (111402ATB, 111403ATB), betaxolol (116801ATB, 116803ATB), bevantolol (117001ATB, 117002ATB), carvedilol (125001ATB, 125002ATB, 125003ATB, 125004ACR, 125005ATB, 125006ACR, 125007ACR, 125007ATR, 125008ACR, 125008ATR), atenolol and other diuretics (262100ATB), metoprolol and felodipine (262400ATR), bisoprolol and thiazides (469800ATB, 469900ATB, 470000ATB), s-atenolol (483101ATB, 483102ATB), nebivolol (489501ATB, 489502ATB, 489503ATB), bisoprolol (117901ATB, 117902ATB), celiprolol (129101ATB), metoprolol (193802ATB, 194003ATR), nadolol (198301ATB), esmolol (154431BIJ, 154430BIJ), labetalol (180230BIJ, 180231BIJ)
C08	amlodipine (107601ATB, 107602ATB, 107603ATB, 459801ATB, 459801ACH, 459802ACH, 459901ATB, 459902ATB, 464601ATB, 470801ATB, 470802ATB, 476201ATB, 479701ATB, 483201ATB, 483202ATB, 486501ATB, 486502ATB, 486503ATB), barnidipine (114001ACH, 114002ACH, 114003ACH), benidipine (115101ATB, 115102ATB, 115103ATB, 115104ATB), cilnidipine (133101ATB, 133102ATB), felodipine (157501ATR, 157503ATR), lacidipine (180301ATB, 180302ATB, 180303ATB), lercanidipine (182001ATB), manidipine (188001ATB, 188002ATB), nicardipine (201003ACR, 201030BIJ, 201031BIJ, 201033BIJ), diltiazem (145703ACR, 145706ATB, 145707ACR, 145707ATR, 145704BIJ), nifedipine (201405ATR, 201409ATR, 528201ATR), nimodipine (201901ATB, 201930BIJ), verapamil (247603ATR, 247605ATR, 247606ATB, 247607ATB, 247630BIJ), nisoldipine (247603ATR, 247605ATR, 247606ATB, 247607ATB),

	dihydropyridine derivatives (441201ATB, 441202ATB)
C09	ACE inhibitors, plain (104201ATB, 104202ATB), candesartan (122601ATB, 122602ATB, 122603ATB), captopril (122901ATB, 122902ATB, 122903ATB), cilazapril (133001ATB, 133002ATB, 133003ATB), enalapril (151601ATB, 151603ATB), irbesartan (177301ATB, 177303ATB), lisinopril (184501ATB), losartan (185701ATB, 185702ATB, 185703ATB), perindopril (211301ATB, 211302ATB), ramipril (222401ATB, 222402ATB, 222404ATB), valsartan (247101ATB, 247102ATB, 247103ATB, 247104ATB), losartan and diuretics (262500ATB, 378900ATB, 486900ATB), valsartan and diuretics (356400ATB, 442600ATB), telmisartan (378801ATB, 378802ATB, 378803ATB), irbesartan and diuretics (385700ATB, 385800ATB), candesartan and diuretics (423700ATB), eprosartan (429201ATB), telmisartan and diuretics (443200ATB, 443300ATB, 502600ATB), ramipril and felodipine (447100ATB, 447200ATB), eprosartan and diuretics (460500ATB), olmesartan medoxomil (468501ATB, 468502ATB, 468503ATB), valsartan and amlodipine (492800ATB, 492900ATB, 495800ATB), olmesartan medoxomil and amlodipine (500500ATB, 582200ATB, 582400ATB, 547600ATB, 547700ATB, 547800ATB, 547900ATB, 548000ATB, 631300ATB, 629400ATB, 629500ATB, 629600ATB, 632800ATB, 632900ATB, 633000ATB), perindopril (501601ATB, 501602ATB), losartan and amlodipine (502700ATB, 503000ATB, 513900ATB, 637400ATB, 637500ATB, 637600ATB), zofenopril (510401ATB, 510402ATB, 510403ATB), telmisartan and amlodipine (511500ATB, 511600ATB, 511700ATB, 623100ATB, 521200ATB, 521300ATB, 521400ATB, 644800ATB, 697300ATB, 697400ATB, 697500ATB, 697600ATB), olmesartan medoxomil and diuretics (513600ATB), fimasartan (515201ATB, 515202ATB, 515203ATB), amlodipine and hydrochlorothiazide (519700ATB, 519800ATB, 520000ATB), fimasartan and diuretics (522000ATB, 526800ATB), valsartan and lercanidipine (522200ATB, 522300ATB, 522400ATB), valsartan and amlodipine (522600ATB, 522700ATB, 522800ATB, 522900ATB, 523000ATB, 523100ATB, 523200ATB, 523300ATB, 523400ATB), perindopril

			and diuretics (556200ATB), fimasartan and amlodipine (651900ATB, 652000ATB, 652100ATB, 652700ATB), candesartan and amlodipine (652900ATB, 653000ATB, 653100ATB), azilsartan medoxomil (662401ATB, 662402ATB, 662403ATB), angiotensin II receptor blockers (ARBs), other combinations (662800ATB, 662900ATB, 663000ATB, 663500ATB, 663600ATB, 663700ATB, 663800ATB, 682700ATB, 682800ATB, 682900ATB), azilsartan medoxomil and diuretics (673500ATB, 673600ATB), valsartan and sacubitril (651401ATB, 651402ATB, 651403ATB)	
		C10BX03–C10BX16	atorvastatin and amlodipine (472300ATB, 472400ATB, 472500ATB, 518900ATB, 614500ATB), rosuvastatin and valsartan (525000ATB, 525100ATB, 525200ATB, 525300ATB, 629700ATB, 629800ATB), rosuvastatin and fimasartan (654700ATB, 654800ATB, 654900ATB, 655000ATB), rosuvastatin and amlodipine (673900ATB, 674000ATB, 674100ATB, 678600ATB)	
	ICD-10	I10-I15		
Heart failure	ICD-10	I42-3, I50, I110, I13		
Dyslipidaemia	ICD-10	E78		ATC code or ICD Diagnosis code
	ATC	C10	acipimox (102201ACH), atorvastatin (111501ATB, 111502ATB, 111503ATB, 111504ATB, 502201ATB, 502202ATB, 502203ATB, 502204ATB), bezafibrate (117101ATB, 117102ATR), fenofibrate (157702ACR, 194901ACH, 194902ACH, 194930ATB, 194930ACH, 520301ACH, 520302ATE), fluvastatin (162401ACH, 162402ACH, 162403ATR), gemfibrozil (165001ACH), lovastatin (185801ATB), pravastatin (216601ATB, 216602ATB, 216603ATB, 216604ATB), simvastatin (227801ATB, 227801ATR, 227802ATB, 227806ATB), rosuvastatin (454001ATB, 454001ATD, 454002ATB, 454002ATD, 454003ATB, 454003ATD, 454005ATB), ezetimibe (462201ATB), pitavastatin (470901ATB, 470902ATB, 470903ATB), simvastatin and ezetimibe (471000ATB, 471100ATB, 507800ATB), pravastatin and fenofibrate (519300ACH), atorvastatin and ezetimibe (633800ATB, 633900ATB, 634600ATB,	

634800ATB), rosuvastatin and ezetimibe (640700ATB, 640800ATB, 640900ATB, 701100ATB), choline fenofibrate (642301ACR, 642301ATR), rosuvastatin and omega-3 fatty acids (663400ACS), Combinations of various lipid modifying agents (679300ACH, 699400ATB, 699500ATB, 702800ATB), omega-3-triglycerides incl. other esters and acids (679401ACS, 679402ACS), atorvastatin and omega-3 fatty acids (694000ACS), colestyramine (132301APD), atorvastatin and amlodipine (472300ATB, 472400ATB, 472500ATB, 518900ATB, 614500ATB), Lipid modifying agents in combination with other drugs (524000ATB, 524100ATB, 527000ATB, 527100ATB, 526300ATB, 526400ATB, 526500ATB, 526900ATB, 644100ATB, 644200ATB, 653200ATB, 629900ATB, 630000ATB, 630100ATB, 630200ATB, 631600ATB, 631700ATB, 634900ATB, 635000ATB, 635100ATB, 635200ATB, 661800ATB, 661900ATB, 662000ATB, 662100ATB, 673700ATB, 663900ATB, 664000ATB, 664100ATB, 664200ATB, 664300ATB, 664400ATB, 671200ATB, 671300ATB, 671400ATB, 671500ATB, 671600ATB, 671700ATB, 677000ATB, 677100ATB, 677300ATB, 677400ATB, 677500ATB, 686800ATB, 686900ATB, 679500ATB, 679600ATB, 679700ATB, 680300ATB, 691400ATB, 691500ATB, 683000ATB, 683100ATB, 683200ATB, 691200ATB, 693000ATB, 684300ATB, 684400ATB, 684500ATB, 684600ATB, 684700ATB, 688100ATB, 688200ATB, 688300ATB, 688400ATB, 688500ATB, 690400ATB, 690500ATB, 690600ATB, 690700ATB, 692000ATB, 692100ATB, 692200ATB, 692300ATB, 692400ATB, 692500ATB, 701800ATB, 701900ATB, 702100ATB, 702200ATB, 706300ATB, 706400ATB, 706500ATB, 706600ATB, 706700ATB), rosuvastatin and valsartan (525000ATB, 525100ATB, 525200ATB, 525300ATB, 629700ATB, 629800ATB), rosuvastatin and fimasartan (654700ATB, 654800ATB, 654900ATB, 655000ATB), rosuvastatin and amlodipine (673900ATB, 674000ATB, 674100ATB, 678600ATB), evolocumab (659501BIJ), alirocumab (674701BIJ, 674702BIJ)

Non-cardiac Comorbidities

Diabetes	ATC	A10	acarbose (100601ATB, 100602ATB), glibenclamide (165402ATB), gliclazide (165602ATB, 165603ATR, 165604ATR), glimepiride (165701ATB, 165702ATB, 165703ATB, 165704ATB), glipizide (165801ATB), metformin (191501ATB, 191502ATB, 191502ATR, 191503ATB, 191503ATR, 191504ATB, 191504ATR, 191505ATR), voglibose (249001ATB, 249002ATB), repaglinide (379501ATB, 379502ATB, 379503ATB), nateglinide (430201ATB, 430202ATB, 430203ATB), pioglitazone (431901ATB, 431902ATB), metformin and sulfonylureas (443400ATB, 443500ATB, 474200ATB, 474300ATB, 474300ATR, 498600ATB, 497200ATB), mitiglinide (486101ATB), metformin and pioglitazone (498100ATB), vildagliptin (500801ATB), sitagliptin (501101ATB, 501102ATB, 501103ATB), metformin and sitagliptin (502300ATB, 502300ATR, 502900ATB, 513700ATB, 513700ATR, 524700ATR), metformin and vildagliptin (507000ATB, 507100ATB, 519600ATB), metformin and saxagliptin (518500ATR, 518600ATR),<br>Combinations of oral blood glucose lowering drugs (518800ATB, 641800ATR, 641900ATR, 642000ATR, 648400ATB, 648500ATB, 648600ATB, 671800ATR, 673800ATR, 671900ATR, 672000ATR, 672100ATR, 672500ATR, 672600ATR, 672700ATR, 672800ATR, 672900ATR, 683300ATR, 683400ATR), metformin and linagliptin (520500ATB, 520600ATB, 520700ATB), metformin and gemigliptin (523800ATR, 632000ATR, 645000ATR, 654100ATR), glimepiride and pioglitazone (525500ATB, 525600ATB), lobeglitazone (525901ATB), dapagliflozin (527302ATB), saxagliptin (613301ATB, 613302ATB), linagliptin (616401ATB), gemigliptin (619101ATB), alogliptin (624202ATB, 624203ATB), teneligliptin (627301ATB), empagliflozin (628201ATB, 628202ATB), pioglitazone and alogliptin (630500ATB, 630600ATB), metformin and alogliptin (635600ATB, 635700ATB, 675500ATB), ipragliflozin (636101ATB), Dipeptidyl peptidase 4 (DPP-4) inhibitors (639601ATB), metformin and dapagliflozin (639800ATR, 641400ATR), evogliptin (645301ATB), metformin and empagliflozin (649000ATB, 649100ATB, 649200ATB, 649300ATB, 649400ATB, 649500ATB), metformin and	ATC code and/or ICD Diagnosis code

			evogliptin (649900ATR, 650000ATR, 650100ATR), metformin and lobeglitazone (653800ATR, 653900ATR, 654000ATR, 655700ATR), gemigliptin and rosuvastatin (664600ATB, 664700ATB, 664800ATB), ertugliflozin (674301ATB, 674302ATB), vildagliptin (685801ATB, 701201ATB), metformin and vildagliptin (702500ATB, 702600ATB, 702700ATB, 704100ATB, 704200ATB, 704300ATB), insulin (human) (170130BIJ, 170131BIJ, 170131BIJ, 170430BIJ, 170431BIJ), insulin lispro (175330BIJ, 175331BIJ, 175332BIJ, 175333BIJ), insulin aspart (441330BIJ, 441331BIJ, 441332BIJ, 441333BIJ), insulin glargine (461830BIJ, 461831BIJ, 461832BIJ), insulin glulisine (484930BIJ, 484931BIJ), insulin detemir (488730BIJ), insulin degludec and insulin aspart (626700BIJ), insulin degludec (626830BIJ), dulaglutide (639701BIJ, 639702BIJ), insulin glargine and lixisenatide (666700BIJ, 667000BIJ), insulin degludec and liraglutide (693900BIJ)	
	ICD-10	E10-11, E14		
Chronic kidney disease	ICD-10	E10.2, E11.2, E14.2, N03, N05, N11.0, N14, N16, N18-N19, N26.9		
	ICD-10	F32-33		
Keratopathy	ICD-10	H17, H18		https://www.aao.org/practice-management/coding/icd-10-cm
Amiodarone	ATC	C01BD01	amiodarone (107401ATB)	
**Charlson Comorbidity Index**				
Myocardial infarction	ICD-10	I21, I22, I25.2		PMID 16224307
Congestive heart failure	ICD-10	I09.9, I11.0, I13.0, I13.2, I25.5, I42.0, I42.5, I42.6, I42.7, I42.8, I42.9, P29.0, I43, I50		
Peripheral vascular disease	ICD-10	I70, I71, I73.1, I73.8, I73.9, I77.1, I79.0, I79.2, K55.1, K55.8, K55.9, Z95.8, Z95.9		
Cerebrovascular disease	ICD-10	G45, G46, I60, I61, I62, I63, I64, I65, I66, I67, I68, I69		
Dementia	ICD-10	F00, F01, F02, F03, G30, F05.1, G31.1		
Chronic pulmonary disease	ICD-10	J40, J41, J42, J43, J44, J45, J46, J47, J60, J61, J62, J63, J64, J65, J66, J67, I27.8, I27.9, J68.4, J70.1, J70.3		
Rheumatic disease	ICD-10	M05, M06, M32, M33, M34, M31.5, M35.1, M35.3, M36.0		

Peptic ulcer disease	ICD-10	K25, K26, K27, K28
Mild liver disease	ICD-10	B18, K73, K74, K70.0, K70.1, K70.2, K70.3, K70.9, K71.3, K71.4, K71.5, K71.7, K76.0, K76.2, K76.3, K76.4, K76.8, K76.9, Z94.4
Diabetes without chronic complication	ICD-10	E10.0, E10.1, E10.6, E10.8, E10.9, E11.0, E11.1, E11.6, E11.8, E11.9, E12.0, E12.1, E12.6, E12.8, E12.9, E13.0, E13.1, E13.6, E13.8, E13.9, E14.0, E14.1, E14.6, E14.8, E14.9
Diabetes with chronic complication	ICD-10	E10.2, E10.3, E10.4, E10.5, E10.7, E11.2, E11.3, E11.4, E11.5, E11.7, E12.2, E12.3, E12.4, E12.5, E12.7, E13.2, E13.3, E13.4, E13.5, E13.7, E14.2, E14.3, E14.4, E14.5, E14.7
Hemiplegia or paraplegia	ICD-10	G81, G82, G04.1, G11.4, G80.1, G80.2, G83.0, G83.1, G83.2, G83.3, G83.4, G83.9
Renal disease	ICD-10	N18, N19, I12.0, I13.1, N03.2, N03.3, N03.4, N03.5, N03.6, N03.7, N05.2, N05.3, N05.4, N05.5, N05.6, N05.7, N25.0, Z49.0, Z49.1, Z49.2, Z94.0, Z99.2
Any malignancy, including lymphoma and leukaemia, except malignant neoplasm of skin	ICD-10	C00, C01, C02, C03, C04, C05, C06, C07, C08, C09, C10, C11, C12, C13, C14, C15, C16, C17, C18, C19, C20, C21, C22, C23, C24, C25, C26, C30, C31, C32, C33, C34, C37, C38, C39, C40, C41, C43, C45, C46, C47, C48, C49, C50, C51, C52, C53, C54, C55, C56, C57, C58, C60, C61, C62, C63, C64, C65, C66, C67, C68, C69, C70, C71, C72, C73, C74, C75, C76, C81, C82, C83, C84, C85, C88, C90, C91, C92, C93, C94, C95, C96, C97
Moderate or severe liver disease	ICD-10	I85.0, I85.9, I86.4, I98.2, K70.4, K71.1, K72.1, K72.9, K76.5, K76.6, K76.7
Metastatic solid tumour	ICD-10	C77, C78, C79, C80
AIDS/HIV	ICD-10	B20, B21, B22, B24

Supplementary Table S2. Comparison of Charlson Comorbidity Index between males and females

	Total (n=188)	Male (n=95)	Female (n=93)	*P* value
CCI scores (points)	3.0 [2.0; 6.0]	3.0 [2.0; 6.0]	3.0 [2.0; 5.0]	0.801
Myocardial infarction	22 (11.7%)	13 (13.7%)	9 (9.7%)	0.530
Onset age (years)	53.8 ± 12.0	49.7 ± 10.3	59.6 ± 12.4	0.055
Congestive heart failure	98 (52.1%)	41 (43.2%)	57 (61.3%)	0.019*
Onset age (years)	48.8 [39.0;55.8]	46.3 [32.5;52.3]	50.9 [44.0;58.6]	0.018*
Peripheral vascular disease	42 (22.3%)	24 (25.3%)	18 (19.4%)	0.425
Onset age (years)	48.1 [35.6;55.6]	44.0 [33.1;50.9]	54.7 [48.2;57.4]	0.009*
Cerebrovascular disease	54 (28.7%)	27 (28.4%)	27 (29.0%)	1.000
Onset age (years)	49.6 ± 10.7	43.9 ± 9.6	55.2 ± 8.6	<0.001*
Dementia	4 (2.1%)	3 (3.2%)	1 (1.1%)	0.628
Onset age (years)	56.2 [49.8; 70.3]	55.7 [49.8;56.6]	70.3 [N/A; N/A]	N/A
Chronic pulmonary disease	123 (65.4%)	60 (63.2%)	63 (67.7%)	0.612
Onset age (years)	44.0 [30.2;54.4]	37.7 [23.5;46.7]	48.6 [40.7;57.9]	<0.001*
Rheumatic disease	23 (12.2%)	10 (10.5%)	13 (14.0%)	0.617
Onset age (years)	47.7 ± 16.5	43.6 ± 17.3	50.9 ± 15.8	0.305
Peptic ulcer disease	93 (49.5%)	46 (48.4%)	47 (50.5%)	0.885
Onset age (years)	42.7 ± 15.6	36.9 ± 15.0	48.4 ± 14.0	<0.001*
Mild liver disease	92 (48.9%)	48 (50.5%)	44 (47.3%)	0.768
Onset age (years)	44.7 ± 14.2	40.5 ± 13.6	49.4 ± 13.5	0.002*
Diabetes without complication	86 (45.7%)	48 (50.5%)	38 (40.9%)	0.237
Onset age (years)	47.1 [36.0;54.7]	42.7 [32.6;51.5]	50.8 [42.8;58.6]	0.003*
Diabetes with complication	4 (2.1%)	3 (3.2%)	1 (1.1%)	0.628
Onset age (years)	46.1 [15.3;53.7]	43.8 [15.3; 53.7]	48.4 [N/A; N/A]	N/A
Hemiplegia or paraplegia	11 (5.9%)	4 (4.2%)	7 (7.5%)	0.511
Onset age (years)	49.1 ± 11.5	50.1 ± 10.7	48.5 ± 12.7	0.837
Renal disease	60 (31.9%)	37 (38.9%)	23 (24.7%)	0.053
Onset age (years)	45.4 ± 11.2	42.5 ± 9.5	50.1 ± 12.4	0.010*
Any malignancy	17 (9.0%)	10 (10.5%)	7 (7.5%)	0.644
Onset age (years)	51.1 ± 11.5	48.5 ± 12.8	54.9 ± 9.1	0.273
Moderate or severe liver disease	1 (0.5%)	1 (1.1%)	0 (0.0%)	1.000
Onset age (years)	48.9 [N/A; N/A]	48.9 [N/A; N/A]	N/A	N/A
Metastatic solid tumour	2 (1.1%)	2 (2.1%)	0 (0.0%)	0.487
Onset age (years)	44.6 [N/A; N/A]	44.6 [N/A; N/A]	N/A	N/A
AIDS/HIV	0 (0.0%)	0 (0.0%)	0 (0.0%)	1.000
Onset age (years)	N/A	N/A	N/A	N/A

Data are presented as n (%) or mean ± standard deviation.

CCI Charlson comorbidity index, *N/A* not available, *AIDS/HIV* human immunodeficiency virus infection and acquired immunodeficiency syndrome.

* <0.05

Supplementary Table S3. Comparison between patients with Fabry disease with and without keratopathy

	Male			Female		
	**Non-keratopathy (n=74)**	**Keratopathy (n=21)**	***P* value**	**Non-keratopathy (n=70)**	**Keratopathy (n=23)**	***P* value**
Age at diagnosis	37.8 ± 15.3	30.8 ± 13.1	0.059	48.8 ± 16.5	45.9 ± 13.3	0.443
Age at start of treatment	37.8 ± 15.1	30.7 ± 13.1	0.056	49.2 ± 16.2	46.0 ± 13.2	0.384
Difference in diagnosis and treatment	0.5 ± 1.1	0.3 ± 0.3	0.368	0.9 ± 1.7	0.6 ± 0.8	0.351
Amiodarone use	3 (4.1%)	1 (4.8%)	1.000	4 (5.7%)	3 (13.0%)	0.484
CCI scores (points)	4.4 ± 3.5	3.5 ± 2.7	0.313	3.7 ± 2.4	3.7 ± 2.2	0.965
Myocardial infarction	10 (13.5%)	3 (14.3%)	1.000	9 (12.9%)	0 (0.0%)	0.161
Onset age (years)	51.9 ± 10.1	42.3 ± 8.4	0.164	59.6 ± 12.4	N/A	N/A
Congestive heart failure	33 (44.6%)	8 (38.1%)	0.779	43 (61.4%)	14 (60.9%)	1.000
Onset age (years)	45.1 ± 13.1	35.3 ± 7.8	0.050*	50.0 ± 14.0	50.5 ± 9.3	0.908
Peripheral vascular disease	19 (25.7%)	5 (23.8%)	1.000	11 (15.7%)	7 (30.4%)	0.213
Onset age (years)	41.4 ± 12.9	39.2 ± 13.3	0.742	49.7 ± 14.3	49.1 ± 17.5	0.936
Cerebrovascular disease	23 (31.1%)	4 (19.0%)	0.421	20 (28.6%)	7 (30.4%)	1.000
Onset age (years)	44.7 ± 9.4	39.5 ± 10.7	0.330	55.7 ± 9.2	53.8 ± 7.2	0.633
MACE	9 (12.2%)	5 (23.8%)	0.327	7 (10.0%)	1 (4.3%)	0.682
Onset age (years)	52.9 ± 8.2	44.1 ± 10.1	0.099	60.8 ± 11.3	54.7 ± N/A	N/A
CV related death	2 (2.7%)	3 (14.3%)	0.123	1 (1.4%)	1 (4.3%)	0.993
Myocardial infarction	3 (4.1%)	1 (4.8%)	1.000	2 (2.9%)	0 (0.0%)	1.000
Stroke	4 (5.4%)	1 (4.8%)	1.000	4 (5.7%)	0 (0.0%)	0.562
Death	10 (13.5%)	3 (14.3%)	1.000	1 (1.4%)	1 (4.3%)	0.993

Data are presented as n (%) or mean ± standard deviation.

CCI Charlson Comorbidity Index, *MACE* major adverse cardiovascular events, *CV* cardiovascular.

* <0.05

Supplementary Figure S1. Study flowchart

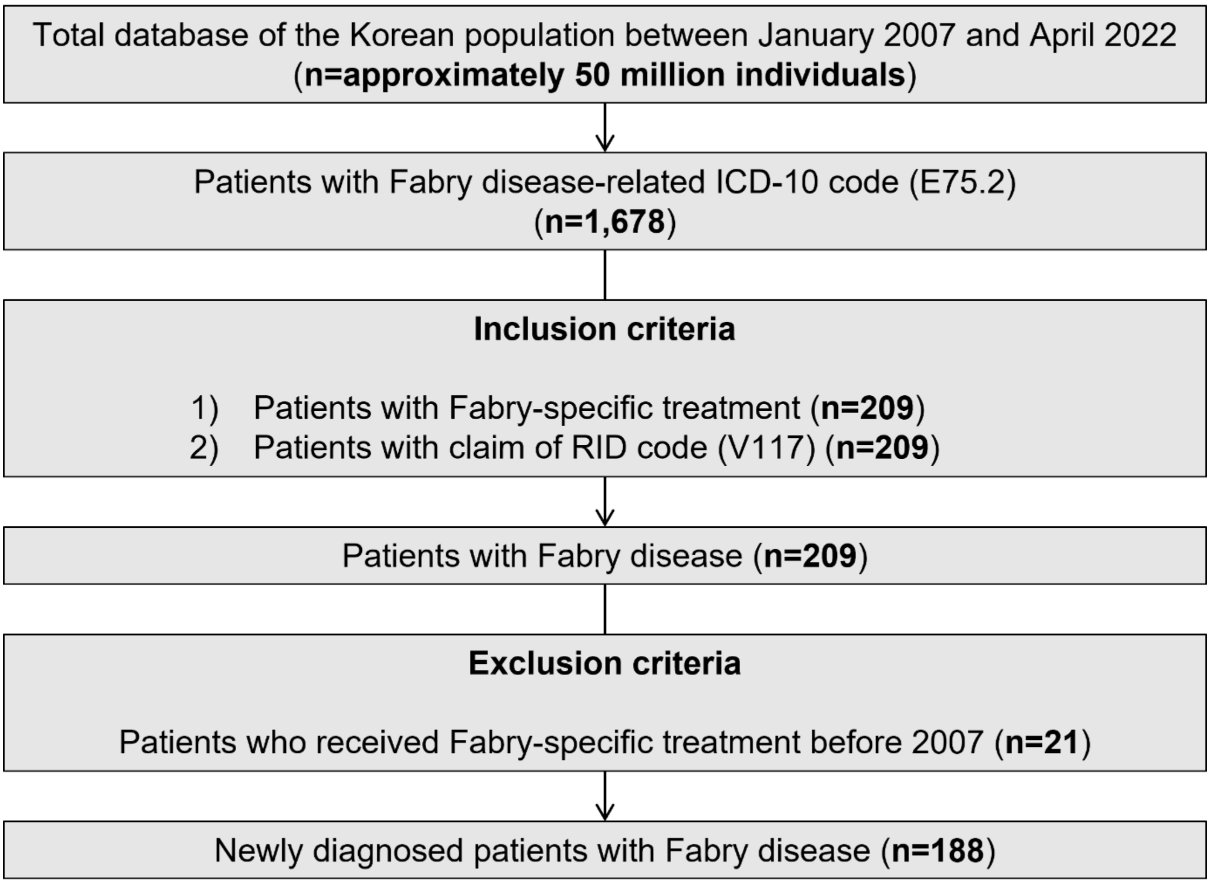

ICD-10 International Classification of Diseases–Tenth Revision, *RID* rare intractable disease registry.
